# Ethical Challenges and Opportunities Associated With the Ability to Perform Medical Screening From Interactions With Search Engines: Viewpoint

**DOI:** 10.2196/21922

**Published:** 2020-09-16

**Authors:** Elad Yom-Tov, Yuval Cherlow

**Affiliations:** 1 Microsoft Research Herzeliya Israel; 2 Faculty of Industrial Engineering and Management Technion Haifa Israel; 3 The Tzohar Rabbinical Organization Lod Israel

**Keywords:** search engines, diagnosis, screening

## Abstract

Recent research has shown the efficacy of screening for serious medical conditions from data collected while people interact with online services. In particular, queries to search engines and the interactions with them were shown to be advantageous for screening a range of conditions including diabetes, several forms of cancer, eating disorders, and depression. These screening abilities offer unique advantages in that they can serve a broad strata of the society, including people in underserved populations and in countries with poor access to medical services. However, these advantages need to be balanced against the potential harm to privacy, autonomy, and nonmaleficence, which are recognized as the cornerstones of ethical medical care. Here, we discuss these opportunities and challenges, both when collecting data to develop online screening services and when deploying them. We offer several solutions that balance the advantages of these services with the ethical challenges they pose.

## Introduction

Recent work has demonstrated the ability to screen for serious medical conditions using search engine logs [1-5]. The development and deployment of these abilities can open new opportunities for earlier diagnosis and more equitable care but require careful consideration of the associated ethical challenges. The goal of this paper is to discuss the ethical pros and cons of these capabilities and to set the stage for a broader discussion of these issues.

Search engines are used by the vast majority of internet users to obtain information on a variety of topics, including medicine [6]. Search engine operators collect information on the interaction of users with their services to improve the operation of their search engines, for example, by measuring user satisfaction from specific answers given to them [7]. It is important to stress that the data collected by search engine operators are not collected to improve medical research or improve people’s health but to enhance search engine operation.

The data collected by search engine operators include, for example, query text, links shown to the user, time of clicking on the links, duration of reading each link, and mouse movements, which serve as a proxy for eye gaze tracking [8]. The data collected by search engines are usually anonymous, in the sense that specific individuals cannot be easily linked to their data, but, unless specified by the user, multiple searches can be attributed, with high likelihood, to the same user.

As noted above, these data have been shown to be effective for screening people for a variety of medical conditions, such as diabetes [4], several forms of cancer [1-3], eating disorders, and depression [5]. Interactions with search engines are useful for such screening because of a combination of factors, including people’s limited knowledge of the association between symptoms and conditions [9]; the fact that many conditions (eg, ovarian cancer) have benign symptoms, of which only the confluence indicates disease, but psychological biases lead people to focus on only the latest symptom [2]; and people’s natural tendency to defer treatment but ask about it online.

These screening capabilities offer unique advantages in that they can serve a broad strata of the society, including people in underserved populations and in countries with poor access to medical services [7]. However, these advantages come at a possible cost to privacy, autonomy, and nonmaleficence, which are recognized as the cornerstones of ethical medical care [10]. Note that the legal aspects of providing (and of not providing) these capabilities are not discussed in this work.

We note that other services, including content providers (eg, Wikipedia and patient groups [11]) and social media platforms (eg, Facebook [12] and Twitter [13]) collect similar data. However, for the reasons described above, we focus on search engines. Moreover, for a broader discussion on the ethics of internet research, readers can refer to the article by Buchanan and Zimmer [14].

In our opinion, the ethical questions that arise from the ability to screen search engine logs should be divided into questions that appear during the development of screening capabilities and questions that should be resolved before medical interventions are provided to people as part of the use of a resulting product. Here, we discuss both these areas.

## Development

### Incidental Finding

“Incidental finding” [15] refers to the case where, during research on one medical topic, data indicates that a person under study has another medical condition of which he/she is (possibly) unaware. Consider a person who contributes their genetic information to build a new screening test for a specific hereditary condition. Upon examination, researchers realize that this person’s genetic information reveals that he/she has another, perhaps common, mutation, which indicates that the person has a serious medical condition that he/she may not know about.

The commonly accepted solution to this challenge in genetic research is to screen for mutations that are common, life-saving, and do not require the person to have a deep understanding of genetics in order to decide whether he/she would like to be treated. If such a mutation is identified, the researcher informs the person that he/she should consult with a genetic counsellor but does not provide advice, as this is not the researcher’s specialty. This route is taken also because, if the burden of treatment (or advice) is placed on the researcher, medical research will, in practice, be severely restricted. This is also the reason that, in many cases, ethics committees recommend opting for completely anonymous research, which reduces the ethical burden on the researcher.

We claim that there is similarity between the question of incidental finding in the medical domain and the case where researchers use data collected during people’s interactions with search engines to later determine that a user may have a medical condition. This can arise from a simple interaction, such as a query suggesting suicidal ideation, to a more elaborate insight obtained from a predictive model based on interactions with the internet service. However, the analogy is not perfect. People who donate their data for medical research know that their data will be examined for medical purposes, whereas people who use a search engine do not expect their data to be used for medical research. In fact, in many cases, people who use search engines may not realize that their interaction data are being collected. We note in passing that routine experiments, such as Facebook’s modification of the order of postings by friends, caused an uproar when they were described in an academic paper [16].

Nevertheless, we argue that these differences should not prevent us from using the insight medical ethics has garnered on the question of incidental findings because people who contribute their data to medical research may not realize that other findings are possible, and on the other hand, as public awareness of search engine data grows, people will realize that these data can provide them with benefits.

### Informed Consent and Autonomy

Search engines, as other internet services, have a system of consent that often includes the use of data collected by the search engine for research purposes. People who use a search engine implicitly consent to its use and further can click on the link at the bottom of the search page where its “Terms of Use” are specified. However, it is difficult to refer to this as informed consent in the medical sense. For example, the authors found that in a sample of approximately 116 million users, only 0.05% clicked on the Terms of Use page during a 1-month period. Experience from other web services suggests that even if a pop-up window would require people to consent to their data being used for research, most people would click on the window without considering what they are consenting to [17].

Additionally, Terms of Use are necessarily broad in their description and, we assume, are often broader than informed consent forms signed by people participating in medical research.

Thus, it is still a challenge to develop a form of consent that both satisfies the ethical requirements for data use and does not overburden users in their interactions with the search engine.

### Willingness to Provide Search Data for Medical Research

There is often an implicit assumption that people would not want their data, collected for other purposes, to be used for medical research without their specific consent. Gefen et al [18] tried to quantify the value that people assign to their data and found that, in a sample of people from around the world, 99% were willing to provide their search engine data in exchange for monetary compensation lower than US $1500 and 53% were willing to pay to have their data analyzed, even if the value of the analysis would be to the society at large rather than to them directly.

Thus, while a minority of users would not agree to the use of their data regardless of compensation, many would agree, and a relevant portion of the population even sees value in the availability of these services, which exceeds that of the data itself.

### Anonymity

As noted above, most of the search data used for medical research has, to date, been anonymous as far as researchers are concerned. This anonymization is provided through the provision of a random user identifier and by not including information that could easily compromise anonymity (eg, location). However, as shown in the AOL leak [19], a malicious researcher may be able to identify a small number of users when such anonymization is used. Therefore, it may be necessary to assume that data are not fully anonymized to a malicious researcher and perhaps even sometimes to a benevolent researcher.

Companies collecting data may, on the other hand, be able to identify a user. This can happen, for example, if users register to their services with their real name. Thus, even if data are anonymous to a researcher, it could, conceivably, be deidentified by the organization collecting the data. In such a case, the problem of incidental findings can arise, as described above.

Finally, an advantage to having data linked to an individual (either anonymous or identifiable) is the ability of users to control the use of their data, as offered, for example, in the European Union’s recent General Data Protection Regulation (GDPR).

### Representativeness

The question of representation in internet data appears in several forms. First, there are the questions of who uses the services from which data are collected and whether they faithfully characterize the entire population. Second, not all people use the internet in similar ways to acquire information, which causes another form of bias in the data.

The first source of representation bias could greatly impact populations, especially in financially disadvantaged parts of the society and in countries with lower access to the internet. Although many efforts have been devoted to closing this gap, it still exists. For example, the percentage of people with access to the internet in different countries ranges from almost 0% to 100% [20]. Thus, it is important to account for such representation biases when using the data to build a model that can be useful to all people.

The second source of representation bias is less well known but is no less important. As shown in past work [21], the use of search engines for medical queries, for example, is highly dependent on people’s age and gender. Moreover, only around 16% of people use search engines to query for medical information [9], adding to the representation bias.

### Summary

Taking the above-mentioned points into consideration, we suggest that in the case of research on medical conditions using search engine data collected for operational purposes, it may be preferable to use anonymized data rather than to obtain consent for the use of identifiable information. If the former route is taken, it is important that ethical committees approve the research, serving in their capacity as representatives of society. We recognize that this is an imperfect solution because of both the inability to seek informed consent and the difficulty for ethics committees to represent search engine users who come from a range of countries and societies, each with its own norms and expectations. However, we view this as a balance between the competing challenges outlined above.

## Production

### Approaches for Providing Search-Based Screening Information

Once a screening method is developed, it may be put into regular use. This could be done in several ways, which are described below.

Suppose an anonymous search engine user is predicted to have a medical condition (eg, screened positive according to interactions with the search engine for the medical condition). The first and most intrusive way to provide the user with this information is to display a notice at a prominent location on the screen. This is currently done only to people who search for information on how to kill themselves [22] or for related topics. In such cases, a banner notice is displayed with the telephone number of a local helpline.

Another way that could be used is to bias (modify) search results toward suggesting the suspected condition. For example, if a user searches for “constant thirst,” instead of showing the regular set of results, users who are predicted to have diabetes will be shown more results that suggest diabetes. A similar “personalized search” is currently part of the service of all major search engine providers (eg, when results are served such that they are relevant to the user’s current location). Therefore, such a solution might not be perceived as a major change by users.

The third way we envision to display information is through the use of search advertisements [3]. Advertisements are not part of the main search results (“organic results”) and are assessed differently by users [23]. People who search for diagnostic information (“do I have diabetes?”) will be shown advertisements that would suggest help in diagnosis (“Worried you have diabetes? Click here to obtain more information”). People who click on the advertisements will be given diagnostic assistance, for example, in the form of clinical questionnaires. As shown recently [3], it is possible to train advertising systems to focus on people who are the most at risk.

We note that advertising in the health domain is currently limited by the policy of advertising systems. On one hand, this prevents abuse of the system by purveyors of unapproved medical services, but also means that any use of this method will often require approval by advertising system managers.

A fourth method to inform people of a possible medical concern is through the normal use of a search engine after first obtaining informed consent to provide these insights. If this method is adopted, users will be shown an informed consent form whenever they are identified as new users by the search engine. The form will offer the users to receive screening information but will default to not receiving the information unless the users positively indicate their interest in receiving this information. Users who consent will then be given alerts whenever a possible medical condition is predicted, based on their queries and behaviors.

Finally, a system might be built where users register and agree to provide their search data on a continuous basis in exchange for alerts when a medical concern is identified in these searches. The data collection, storage methods, and data use would be clearly described to the user. This is similar to services that analyze people’s genetic material, where their data will be the search data (or browsing data, in general) and the analysis will be conducted on an ongoing basis, rather than a single transfer of data. Such a system could be offered by search engine providers or, perhaps preferably, by medical providers or dedicated companies.

### Unsolicited Diagnosis

Unsolicited diagnosis [24] or unsolicited medical opinion [25] refers to the case where people may be provided with medical information when they do not expect it. For example [26], consider the case of a dermatologist who is standing at the back of an elevator at the mall and notices that the person standing in front has a mole that the dermatologist thinks is likely cancerous. In this case, the person who has the mole is not expecting to receive a diagnosis from a random person at the mall (though a specialist in this case), and thus, this is a case of unsolicited diagnosis. Medical ethicists have considered the question of whether the medical specialist has a duty to inform the person to seek medical attention and whether the specialist has a right to do so. On one hand, the person is not expecting a diagnosis and there is no doctor-patient relationship between the two. On the other hand, not informing the person may lead to serious and irreparable damage. The conclusion reached by some ethicists [26] is that medical doctors have a duty to offer their unsolicited medical opinion, especially when the medical condition requires urgent attention for treatment. However, doctors need to consider the possible harm of such an intervention. Note that a legal duty to act is very much country-specific, often defined through legislation (ie, “good Samaritan” laws [27]) to protect people who take such action.

We note in passing that the balance between benefit and harm for the individual may differ from that for the society. For example, some conditions currently have no treatment because they cannot be identified early enough and so many people would prefer not to know that they have such conditions. However, suppose search engine data could provide such an early alert [28]. In such a case, if enough people knew they have these conditions, pharmaceutical companies might be compelled to develop treatments. However, as this is a secondary effect, we have not focused on it.

### Risk Compensation

Risk compensation (also referred to as moral hazard [29]) describes increased risk taking caused by the perceived usefulness of safety measures. For example, it has been suggested that condom distribution fosters inhibition among HIV-positive people [30].

If internet platforms disclose offering screening services, users may choose to modify their behaviors in ways that could harm them. For example, as noted above, only around 16% of users queried about medical symptoms prior to diagnosis [9]. It is difficult to predict illness for people who do not query, but they may assume that a screening model is examining their queries and will alert them when it is necessary to visit a health provider, thus preferring not to access medical care even when they think they should. This is especially likely in the fifth solution described above, because users who register with a dedicated service expect it to provide such alerts. Therefore, it may be important for such a service to alert users about its inability to screen when it predicts that they will not ask relevant questions.

### Cost of Errors

No system is perfect, including those discussed in this paper. The cost of errors is an important factor in whether and how information should be provided to users. A false positive error means that a person is informed (depending on the method of provision described above) of a medical condition when he/she does not have one. This can cause undue stress and result in unnecessary medical procedures [31]. A false negative error means that a person who should have been provided with an alert does not receive one, possibly causing late diagnosis (as described above).

### Summary: Advantages and Disadvantages of Different Notification Methods

The first method described above, whereby a notice is shown to the user, is advantageous in that it provides people with immediate, clear, and actionable information. However, we advocate its use in only the most extreme situations (eg, expressed intention of suicide) because it is intrusive and may cause more harm than good in the form of breaching privacy and impinging on people’s autonomy.

The second (biasing results), third (advertisements), and fourth (explicit prior informed consent) methods are advantageous in that they do not force information upon users and allow users to decide if they would like to use the offered information. These methods (especially biasing results and advertisements), however, somewhat impinge on privacy and autonomy. Additionally, not all users will recognize the help offered to them, and only some will make use of it even when they recognize it. We note that in the case of advertising, the act of choosing to click on the advertisement should be considered informed consent (assuming that the advertisement is appropriately phrased). We also recognize that obtaining explicit informed consent (eg, the fourth method) can be difficult from a design perspective and can burden users who, for example, use private browsing. For these reasons, we believe that these methods, especially the one using advertisements, correctly balance benefit and harm.

Finally, the fifth method (dedicated system) is clearly superior in terms of autonomy and consent, but based on prior experience, we assume that only a minority of users, probably skewed toward the more affluent parts of society, will use this method. Thus, while extremely beneficial for individuals, it should be considered less useful at the societal level.
